# Microbial Ecology and Nutritional Features in Liquid Sourdough Containing Hemp Flour Fermented by Lactic Acid Bacterial Strains

**DOI:** 10.3390/foods14030469

**Published:** 2025-02-01

**Authors:** Mariaelena Di Biase, Daniel Scicchitano, Francesca Valerio, Stella Lisa Lonigro, Valentina Cifarelli, Giorgia Ostante, Isabella D’Antuono, Marco Candela, Massimo Ferrara

**Affiliations:** 1 Institute of Sciences of Food Production, National Council of Research, 70126 Bari, Italy; francesca.valerio@cnr.it (F.V.); stellalisa.lonigro@cnr.it (S.L.L.); valentina.cifarelli@ispa.cnr.it (V.C.); giorgia.ostante@ispa.cnr.it (G.O.); isabella.dantuono@cnr.it (I.D.); massimo.ferrara@cnr.it (M.F.); 2 Unit of Microbiome Science and Biotechnology, Department of Pharmacy and Biotechnology, University of Bologna, 40126 Bologna, Italy; daniel.scicchitano2@unibo.it (D.S.); marco.candela@unibo.it (M.C.); 3 Fano Marine Center, 61032 Fano, Italy

**Keywords:** *Lactiplantibacillus plantarum*, *Weissella cibaria*, fermentation products, hemp flour, bioactive molecules, protein profile, EPS, metabarcoding, microbiota

## Abstract

Hemp seed flour (*Cannabis sativa*) is a non-traditional matrix alternative to wheat for baked goods production. The aim of this study was to investigate the microbiota of two liquid sourdoughs (SLs) based on hemp or a wheat–hemp mixture, before and after spontaneous or piloted fermentation (*Lactiplantibacillus plantarum* ITM21B or *Weissella cibaria* C43-11 used as starters). Culture-dependent and -independent (high-throughput sequencing of bacterial phylogenetic V3-V4 regions of the 16S rRNA gene) methods, were used to evaluate the microbial community. The effect of fermentation on the content of bioactive molecules (polyphenols, organic acids, proteins, and amino acids) was also investigated. Results indicated that the microbial community of all SLs was mainly (99.7 ÷ 100%) composed of *Firmicutes* and *Proteobacteria*, and the latter was the unique phylum before fermentation in formulations produced exclusively with hemp flour. Two PCoA plots (Test adonis with pseudo-F ratio, *p* > 0.05) showed no significance difference between the microbial communities of the formulations. However, the relative abundance variation at the family level in the wheat–hemp-based mixture SLs showed a significant enrichment of the *Lactobacillaceae* family (Kruskal–Wallis test, *p* = 0.04). Moreover, results confirmed hemp seed flour as a suitable fermentation substrate to obtain microbial consortia allowing for an increase in organic acids, especially lactic acid (9.12 ± 1.22 and 7.45 ± 0.75 mmol/kg with *Lpb. plantarum* and *W. cibaria*, respectively), in both piloted fermentations, and in polyphenols by 21% and amino acids by 158% in SL fermented by the C43-11 strain.

## 1. Introduction

The use of hemp seed flour (*Cannabis sativa* subsp. *sativa*) as an alternative matrix to wheat to produce sourdough was exploited since it positively influences the antioxidant properties, protein content, and structural characteristics of bread products [1]. Hemp seeds are a rich source of proteins (they contain approximately 20–25% proteins), contain all the necessary amino acids, and are also a rich source of bioactive compounds from the polyphenol group, which have antiallergic, antiatherogenic, anti-inflammatory, antimicrobial, antiviral, antitumor, and cardioprotective effects [2,3]. The lipid portion (25–35%) is essentially made up of polyunsaturated fatty acids (PUFAs) [2]. Hemp flour is rich in vitamin E and minerals [2,4,5], and it has also been used for gluten-free bread-making [6,7].

The use of sourdough in bread production has advantages such as prolonging shelf-life, enhancing bread flavor, improving dough structure, and increasing the nutritional value [8]. The microbiota of a stable sourdough principally consists of lactic acid bacteria (LAB) and yeasts [9]. Generally, microorganisms from the ingredients are transferred to the fermenting flour–water mixture, and their survival depends on the competitiveness toward particular microbial species that can survive in the harsh conditions of the sourdough ecosystem. Moreover, their survival and growth are also determined by the presence of appropriate nutrients and whether or not they are carried over by substrates included in the formulation [10].

The manufacturing of sourdough includes a complex microbial community, and unstandardized fermentation conditions enlarge this community with a consequent effect on the sensory, taste, or aromatic characteristics of the final product [9]. In this context, investigations into the microbiota dynamics occurring during or after fermentation have a crucial importance in discovering the possible connection between the microbiota structure and metabolic profile for optimizing the inoculation of a fermented ingredient in order to improve the food quality of baked goods. Some authors [11] investigated the succession of microbiota during spontaneous and inoculated (with two different microbiota groups) fermentations of Chinese liquor and predicted the final metabolic profiles. Their results demonstrate that microbial inoculation with functional genera plays an important role in microbial metabolism. However, how microbial succession and the final level of desired metabolites were affected is still unknown. To overcome the drawbacks in fermented product manufacturing, especially regarding standardizing the processes, the concept of synthetic microbial communities (SynComs) as a novel strategy to enhance quality, described as a co-culture system established by two or more microorganisms, was introduced [12]. An overview on the SynComs was reported by Jin et al. [13] and showed that their constitution aimed to simplify complex microbial communities and allow for positive interactions for the production of targeted metabolites in food processing.

Some authors reported the identification and characterization of LAB communities occurring in hemp seeds or hemp seed-based sourdough [14,15] to design novel functional starter strains suitable for this flour. Miragoli et al. [14] identified *Pediococcus pentosaceus*, *Leuconostoc mesenteroides*, *Enterococcus mundtii*, and *Enterococcus faecium* from hemp seed, and Nionelli et al. [15] isolated *P. acidilactici*, *Lactiplantibacillus plantarum*, *P. pentosaceus*, and *Leuconostoc mesenteroides* from hemp sourdough. *Lpb. plantarum* is the most common species found in the fermented food ecosystem including fermented cereals. This can be explained by *Lpb. plantarum*’s ability to produce transporters and enzymes involved in carbohydrate fermentation [16]. Heperkan et al. [17] isolated a *Lpb. plantarum* (B2) strain with high β-galactosidase activity, an absence of protease, and strong peptidase activities from boza. In previous studies, liquid sourdoughs produced using *Lpb. plantarum* ITM21B as a starter with a fermentation time of 14 h were successfully integrated in a yeast-leavened bread-making process, leading to a final product with reduced salt content and appreciated sensorial qualities [18,19,20]. To optimize the fermentation conditions necessary to obtain suitable nutritional and technological characteristics, the modeling of growth and organic acid kinetics and the evolution of the protein profile and amino acid content during *Lpb. plantarum* ITM21B fermentation in liquid sourdough was developed [21]. Therefore, considering the features of the *Lpb. plantarum* strain ITM21B, it was investigated in the current study as a starter.

The well-characterized *W. cibaria* strain C43-11 [22] was also included in this study as a producer of dextran, a homopolysaccharide which can be used in foods since it is “generally recognized as safe” (GRAS) by the Food and Drug Administration. The technological importance of dextran is due to its ability to improve the rheology and consistence of many fermented and baked food products, replacing gelling agents or fatty substances used as thickeners to positively influence the viscoelastic properties of dough and the texture and shelf life of bread [23,24,25,26,27,28]. Furthermore, dextran also confers functional properties to the product, acting as prebiotic substance with beneficial effects on human health (antioxidant activity, cholesterol reduction, and possible immunomodulatory and anti-tumor activity) [29,30,31]. Actually, the *W. cibaria* species is not yet recognized as a potential starter admitted in food fermentation since it is not included in the QPS (Qualified Presumption of Safety) list [32]. However, in order to assess the suitability of microorganisms for QPS status, it is fundamental to enlarge the body of knowledge about the use on this species [33]. Moreover, the *Weissella* genus is extensively involved in spontaneous fermentation together with *Carnobacterium*, *Enterococcus*, lactobacilli, *Lactococcus*, *Leuconostoc*, *Oenococcus*, *Pediococcus*, *Streptococcus*, *Tetragenococcus*, and *Vagococcus* [34], is a common inhabitant of the human intestine [35], and its potential use as probiotics [36] has been suggested.

The aim of this study was to investigate the influence of hemp flour and spontaneous fermentation on liquid sourdough compared to piloted (*Lpb. plantarum* ITM21B or *W. cibaria* C43-11) fermentation. Nutritional and technological characteristics, the production of bioactive compounds, including organic acids, polyphenols, amino acids, and exopolysaccharides, and the evolution of sourdough microbiota were investigated.

## 2. Materials and Methods

### 2.1. Bacterial Strains

To produce the SL, two strains belonging to the Collection of the Institute of Science of Food Production, National Research Council (Italy), were used. Two LAB strains were selected in this study: the proteolytic strain *Lpb. plantarum* ITM21B isolated from sourdough [37] and the *W. cibaria* C43-11 strain, which was isolated from wheat semolina and is a producer of exopolysaccharides (EPSs), in particular dextran [20,22], which is already used in the production of Focaccia bread with reduced fat content [23]. For long-term storage, stock cultures (800 µL of a culture in de Man Rogosa and Sharpe (MRS) broth (Conda, Madrid, Spain) with 200 µL of Bacto glycerol (Fisher Scientific UK Ltd., Leicestershire, UK)) were prepared and frozen at −80 °C. To obtain fresh cultures, the strains were subcultured twice (1% vol/vol) in MRS broth for 24 h before use.

### 2.2. Liquid Sourdoughs Preparation

In the current study, two optimized liquid sourdough formulations, deriving from ongoing and previous studies [19,20,22] finalized to assess their applicability in bakery products, were investigated in fermentation experiments. Sourdough formulations were produced and were characterized by a different ratio between hemp flour and wheat flour: SL_C was a mixture of wheat flour and hemp flour in a 1:1 *w*/*w* ratio, while SL_W contained hemp flour alone (Table 1). Both formulations were spontaneously fermented or inoculated with a specific strain: SL_C was inoculated with *Lpb. plantarum* ITM21B, and SL_W was inoculated with *W. cibaria* C43-11 at an expected initial load of 5.00 and 8.00 log CFU/g of the total weight of the SL, respectively. The starting conditions of the fermentation are reported in Table 1. Three biological replicates were performed for each SL and analyzed in duplicate (3 × 2).

At different time intervals, SL samples were collected for microbiological analysis, determination of the pH, total titratable acidity (TTA), organic acids (lactic, acetic, and propionic acids), total amino acid (TFAA) content, L-glutamic acid, EPS and total protein content, and modification of the total protein pattern, and total DNA extraction.

### 2.3. Physicochemical and Microbiological Analyses During Fermentation

At each sampling time, samples were analyzed for LAB cell density as follows: 10 g of SL were diluted (1:10) with sterile 0.1% Bactopeptone (Conda), homogenized for 2 min in a stomacher, and, after a 30 min rest, 100 µL aliquots of serial decimal dilutions in sterile NaCl (0.85% *w*/*v*) + Tween 80 (0.025%) were spread on MRS (Conda) plates and incubated for 48 h at 37 °C. The total LAB count was expressed as log CFU/g of the total weight of the SL. To monitor the presence of the starter strains, at each sampling time, 20% of the total colonies were randomly picked from countable MRS agar plates, isolated, and checked for purity as reported by Di Biase et al. [18].

Bacterial DNA from presumptive isolated LAB colonies was extracted from overnight cultures grown in MRS broth at 37 °C and analyzed by REP-PCR using the primer pair REP-1R-Dt/REP-2R-Dt, as previously described by De Bellis et al. [38]. The amplification products were separated by Lab-on-a-Chip (LoaC) microfluidic electrophoresis using the DNA7500 LabChip Kit and the Bioanalyzer2100 platform, and data were analyzed using the 2100 Expert software (Agilent Technologies, Waldbronn, Germany). The DNA7500 Ladder was used as sizing standard and as a normalization reference, in addition to lower (50 bp) and upper (10.380 bp) markers added to each DNA sample on the chip. The identification of *Lpb. plantarum* ITM21B and *W. cibaria* C43-11 was based on the comparison of the REP-PCR profile of each isolate with the specific pattern obtained from pure cultures of each strain. The reproducibility of fingerprints was verified by repeating the analysis twice.

The pH of the SL was recorded at each sampling time with a portable pH meter (type 110, Eutech Instruments, Singapore City, Singapore) supplied with the Double Pore D electrode (Hamilton, Bonaduz, Switzerland). TTA was measured according to AOAC method no. 981.12 [39] and expressed in mL of 0.1 N NaOH required to achieve a pH of 8.3.

#### 2.3.1. Total Free Amino Acids and Protein Content and Profile in SLs

The SL samples were prepared according to the method originally described by Osborne [40] and modified by Weiss et al. [41] to obtain water/salt-soluble extracts used to quantify TFAAs. TFAAs were determined by the Cd-ninhydrin method, as reported by Doi et al. [42]. Briefly, a solution containing CdCl and ninhydrin was used as a reagent by measuring the absorbance of the sample at 505 nm. TFAA quantification was then carried out using glycine as a standard.

Total proteins were extracted from SL samples (during fermentation time) under reducing conditions. Briefly, an amount of SL samples, weighed on the basis of their flour content and equivalent to 40 mg of flour, was mixed with 200 µL of an extraction solution containing TRIS-HCl 62.5 mM pH 8.8, 5% mercaptoethanol, 2% SDS, and 10% glycerol [43] for 3 h at 4 °C. Mixtures were centrifugated (12,000× *g*, 15 min, and 4 °C), and the insoluble material was removed; afterwards, supernatants, after heating at 100 °C for 2 min, were stored at −20 °C for analysis [20]. The protein concentration was determined by the Bradford method [44]. The Bio-Rad dye reagent (Bio-Rad Laboratories, Hercules, CA, USA) with bovine serum albumin was used as the standard, and total protein quantification was expressed as mg of protein per 500 g of SL.

Total protein extracts were analyzed by LoaC microfluidic electrophoresis using the Protein 230 LabChip kit that explores proteins in the 14–230 kDa molecular weight range and the Bioanalyzer2100 platform (Agilent Technologies, Waldbronn, Germany). Sample preparation and chip loading were performed according to the manufacturer’s instructions. Data analysis was performed by the 2100 Expert software (B02.05 version; Agilent Technologies, Waldbronn, Germany) that employs internal standards to align sample proteins to a molecular weight ladder. Sample data are automatically displayed as peaks (electropherogram), bands (gel-like image), and tables, reporting, for each protein peak, the molecular weight (Mw), time-corrected peak area (TCA), relative concentration (RC), and protein percentage (%) calculated on the total peak areas in the sample. A manual integration of peaks was performed after each run, and low peaks (RC < 20 ng/µL) were excluded from the analysis. Based on molecular weight (Mw), 3 different areas were identified in the electropherograms: A1 (14–30 kDa), A2 (31–79 kDa), and A3 (>80 kDa), and data for each Mw area were merged. Results are the average of 2 experimental replicates for each sample (n = 2).

#### 2.3.2. Extraction and Quantification of Organic Acids in SLs

For the quantification of lactic, acetic, and propionic organic acids in SLs, the high-pressure liquid chromatography method reported in Valerio et al. [20], with slight modification, was used. Samples were diluted 1:10 with 0.15 M H_2_SO_4_, centrifuged (9000× *g*, 20 min, and 2 °C) and ultrafiltered through 3 kDa cut-off micro-concentrators (Vivaspin 6 MWCO, Cytiva, Uppsala, Sweden). Organic acids were separated using an HPLC system (AKTABasic10, P-900 series pump, Amersham Biosciences AB, Uppsala, Sweden), using a Rezex ROA-organic acid H^+^ (8%) column (7.80–300 mm, Phenomenex, Torrance, CA, USA) and UV detector (210 nm). H_2_SO_4_ 0.007 N was used as the mobile phase (Fluka, Deisenhofen, Germany) pumped at a flow rate of 0.7 mL/min (65 °C). The organic acids amount was determined by integrating calibration curves obtained from the relevant standards. Detection limits (LOD) of lactic, acetic, and propionic acids were 0.23 µmol/mL, 2.2 µmol/mL, and 0.51 µmol/mL, respectively. LOQ values corresponded to 2 × LOD. Final concentrations of each organic acid in the liquid sourdough were calculated considering the concentration and/or dilution factors and expressed as mmol/kg or μmol/kg of product.

#### 2.3.3. Extraction and Quantification of L-Glutamic Acid in SLs

The L-glutamic acid content was quantified in all SLs initially diluted (1:2) with distilled water, centrifuged (15,000× *g*, 20 min, and 4 °C), and ultrafiltered through a 3 kDa cut-off micro-concentrator (Microcoon, Amicon Ultra, Merck Millipore, Cork, Ireland). This amino acid was quantified separately as it is involved in compensating for flavor perception. The spectrophotometric quantification method was carried out using the Megazyme kit (K-GLUT 07/12; Megazyme International, Bray, Ireland) following the procedure indicated by the manufacturer.

#### 2.3.4. Extraction and Quantification of EPS in SLs

The production of EPS after fermentation was investigated since these compounds possess different technological and functional properties. Only the SL samples prepared using the starter strain *W. cibaria* C43-11, characterized by its high capacity to produce EPS [20,22], were tested.

For the EPS quantification assay, SL_W samples were diluted with distilled water (1:2) and centrifuged (8000× *g*, 10 min, and 4 °C), and the supernatants were treated with trichloroacetic acid (VWR Chemicals BDH Prolabo, Australia) up to a final concentration of 4% for 2 h at 4 °C, before centrifugation (20,000× *g*, 10 min, 4 °C). Afterward, three volumes of chilled 96–99% (*v*/*v*) ethanol were added to the supernatants, and the solutions were stored overnight at 4 °C. The EPS precipitate was collected by centrifugation (8000× *g* for 20 min at room temperature), dissolved in distilled water, dialyzed (12–14 kDa) against distilled water at 4 °C for 48 h, freeze-dried, and rehydrated with distilled water up to the initial volume. The EPS concentration (g/kg) was determined according to the phenolsulfuric method [45], using glucose as the standard (LOD 0.078 g/kg). Briefly, 500 µL of phenol 5% and 2.5 mL of concentrated H_2_SO_4_ were rapidly added to 500 µL of the sample. The absorbance was measured after 10 min rest and shaking before readings were conducted at 490 nm.

#### 2.3.5. Extraction and Quantification of Total Polyphenols in SLs

To evaluate the potential enrichment in bioactive molecules (polyphenols) of inoculated and non-inoculated SL samples (SL_C and SL_W) and their stability over time, at times 0 and 14 h, the analysis of total polyphenols was carried out. The extraction was performed on the freeze-dried samples by an ultrasonic extraction followed by an orbital shaker extraction with an 80% methanol solution; extraction was repeated twice with fresh solvent, and extracts were centrifuged and filtered. The supernatants were combined and analyzed spectrophotometrically following the method reported in D’Antuono et al. [46], and results are expressed as mg of Gallic Acid Equivalent (GAE) per 100 g of fresh product. Measurements were made in triplicate.

### 2.4. Metabarcoding Analysis

Aliquots of each SL were sampled for total DNA extraction using the PowerSoil DNA Isolation Kit (Qiagen, Hilden, Germany). The DNA samples were quantified using NanoDrop ND-1000 (NanoDrop Technologies, Wilmington, DE, USA) and subsequently processed for sequencing of the V3–V4 hypervariable region of the bacterial 16S rRNA gene. Specifically, this region was amplified by PCR in a reaction volume of 50 µL containing 25 ng of total microbial DNA, 2X KAPA HiFi HotStart ReadyMix (Roche, Basel, Switzerland), and 200 nmol/L of primers 341F and 785R, having, inside them, the sequences of the Illumina adapters for sequencing. The PCR reaction consisted of 3 min at 95 °C, 25 cycles of 30 s at 95 °C, 30 s at 55 °C, 30 s at 72 °C, and a final elongation step at 72 °C. The PCR products were subsequently purified using Agencourt AMPure XP magnetic beads (Beckman Coulter, Brea, CA, USA). The libraries were then obtained through a PCR reaction consisting of a limited number of cycles, using Nextera technology, and subsequently purified using Agencourt AMPure XP magnetic beads. The libraries obtained were combined at a concentration of 4 nM. The pool obtained was denatured using 0.2 N NaOH and diluted to a final loading concentration of 4.5 pM with the 20% PhiX control. Finally, sequencing was performed on the Illumina MiSeq platform using a 2 × 250 bp paired-end protocol, following the manufacturer’s instructions (Illumina, San Diego, CA, USA). For metabarcoding analysis, data were processed through a pipeline that combined PANDAseq [47] and QIIME2 [48]. The “fastq filter” feature of the Usearch11 algorithm [49] was used to keep high-quality reads (min/max length = 350/550 bp). Reads with an expected error per base *E* = 0.03 (i.e., three expected errors every 100 bases) were rejected using the phred Q score probability. The retained reads were subsequently binned into ASVs with DADA2 [50]. Taxonomic assignment was carried out using the VSEARCH algorithm [51] and the SILVA database (December 2017 release) [52]. Sequences ascribed to eukaryotes (including mitochondrial and chloroplast sequences) or left unassigned were eliminated. All samples were normalized to the fewest number of reads possible. Beta diversity was assessed for bacterial communities using unweighted and weighted UniFrac distances.

### 2.5. Statistical Analyses

Data are presented as mean values ± the standard deviation of the mean. Data relevant to sourdough samples (bacterial count, TTA, pH, total polyphenols, L-glutamic acid, TFAA, total protein, and organic acid contents, and EPS only for SL_W) were analyzed by one-way ANOVA followed by Tukey’s test. Results were considered statistically significant at *p* < 0.05. Statistical analysis was performed using Statistica 10.0 (StatSoft Inc., Tulsa, OK, USA). Data separation in the Principal Coordinates Analysis (PCoA) was tested by a permutation test with pseudo-F ratios (function “Adonis” in the “vegan” package of R). The Wilcoxon Rank-Sum test or Kruskal–Wallis test were used for differential analysis, where a *p* ≤ 0.05 was considered as statistically significant.

## 3. Results and Discussion

Cereal fermentation for producing sourdough represents an old biotechnological process to manufacture baked goods. In the case of traditional sourdoughs, the spontaneous fermentation method is mainly adopted, even if the process can be also piloted by starter strains in a single step in the case of industrial sourdoughs [53]. Generally, wheat flour is used as cereal matrix, although flours from pseudocereals (e.g., amaranth, buckwheat, and quinoa), legumes (e.g., beans, chickpeas, lentils, and lupine), and alternative seeds (e.g., acorn, chestnut, chia, flaxseed, hempseed, and sunflower) have been used [53]. Investigations on the microbial communities occurring in alternative sourdoughs give information about the microbial consortia dominating and/or competing with starters during fermentation. In fact, the flour and the microbial starters play key roles in relation to nutrient availability and physico-chemical parameters in the microbial consortia composition [54].

### 3.1. Microbiological and Physico-Chemical Parameters in SLs

The SLs were characterized by their physico-chemical characteristics (pH and TTA), LAB cell density, and nutritional profile at two fermentation times (t0 and t14; SL_C and SL_W). Data are reported in Table 2.

Microbial count after fermentation showed that hemp seeds flour was a suitable substrate for LAB growth. During fermentation, the acidification process led to a pH decrease and TTA increase. Among SL_W samples, the highest acidification was observed in SL_W C43-11 in comparison to spontaneously fermented SL_WS. The formulation SL_C showed a similar pH in spontaneous and piloted fermentation, even if the TTA value was significantly higher in the presence of the starter strain ITM21B. Acidification induced by LAB in sourdough modulates the activity of cereal enzymes and of naturally occurring microorganisms [55]. Čižeikienė et al. [56] found that the biochemical properties of the hemp seed-based fermentation products strictly depended on the metabolism of the LAB strain used.

**Figure 1:**
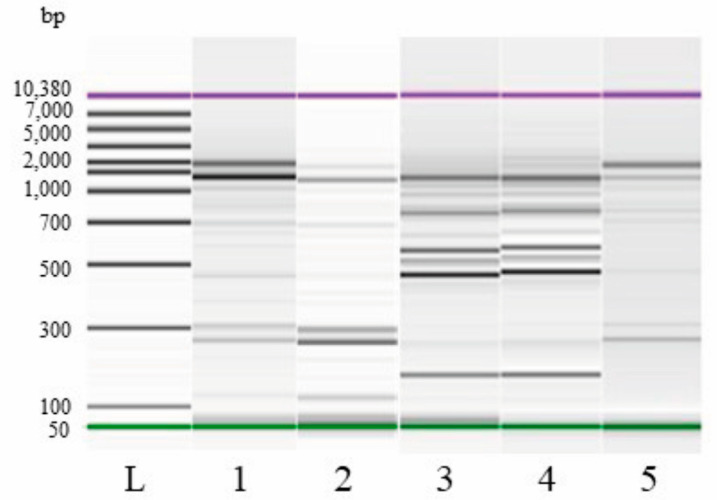
REP-PCR profiles, presented as a gel-like image on DNA7500 LabChip, of *W. cibaria* strain C43-11 (1) and *Lpb. plantarum* strain ITM21B (3) and of representative profiles of colonies isolated from SL_W C43-11 (2) and SL_C 21B (4, 5) at t14; molecular weight ladder (L).

In SL_C 21B and in SL_W C43-11 at t14, the presence of the starter strains was monitored by comparing the REP-PCR profiles of each isolate with the specific pattern of their pure cultures. In Figure 1, the electrophoretic profiles, presented as a gel-like image on DNA7500 LabChip, of the starter strains *W. cibaria* C43-11 (lane 1) and *Lpb. plantarum* ITM21B (lane 3) are reported. The comparison of profiles of all isolates from SL_W C43-11 delineated only one banding pattern (lane 2) corresponding to the REP-PCR profile of the starter strain (lane 1). In the case of SL_C21B, two different banding patterns were found after fermentation. Moreover, 77.8% of the analyzed colonies presented a REP-PCR profile (lane 4) that was perfectly superimposable to the profile of the pure culture of the starter strain (lane 3), while the remaining 22.2% presented a different banding pattern (lane 5).

The fermentative activity of the consortia was demonstrated by the presence of organic acids in all the liquid sourdoughs (Table 3).

The fermentation process resulted in an increase in lactic acid in all SL formulations obtained by spontaneous or piloted fermentation. Among SL_C samples, formulation SL_C 21B showed the highest content of lactic and acetic acids, as also demonstrated in similar studies involving strain ITM21B as a sourdough starter [19,45]. Considering the SL_W samples, only the content of lactic acid significantly increased when strain C43-11 was used as a starter. Other researchers have studied the effect of LAB on lactic acid formation in fermented hemp seed products. The study of Čižeikienė et al. [56] confirmed the ability of a LAB strain to produce lactate in hemp-based fermented products, and they found a high lactic acid content, ranging between 103 and 133 mmol/kg, after 48 h by Lactobacillus acidophilus DSM 20079 or *Lpb. plantarum* MR24 or Levilactobacillus brevis R26 strains. Although our results showed less content after a short fermentation (14 h), the data confirmed the production of metabolites that were strictly strain-related and dependent on the fermentation conditions.

Table 4 shows the total protein content in all SL formulations at sampling times t0 and t14. In both piloted SL samples and in SL_WS, the protein content did not show significant changes, while spontaneous fermentation in SL_CS caused a significant reduction.

**Figure 2:**
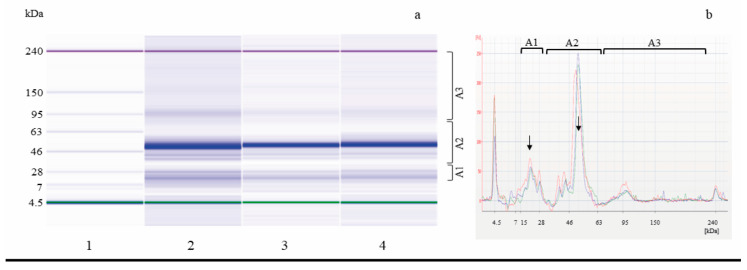
Electrophoretic analysis (LoaC) of total proteins from SL_C inoculated with *Lpb. plantarum* ITM21B (SL_C 21B) or not (SL_CS) shown as gel-like images (**a**) and overlaid electropherograms (**b**) on Protein230 LabChip. Ladder, weight marker (1), SL_CS t0 (2, red line), SL_CS t14 (3, blue line), and SL_C 21B t14 (4, green line). Brackets indicate molecular weight areas: A1 (14–30 kDa), A2 (31–79 kDa), and A3 (80–230 kDa); arrows indicate predominant 17.0 and 53.0 kDa protein peaks/bands.

To highlight changes in the protein pattern of fermented samples, microfluidic electrophoresis was performed. Figure 2 shows representative electrophoretic profiles of the total proteins extracted from SL_C liquid sourdoughs fermented with hemp/wheat flour.

At sampling time t0, approximately 10 peaks/bands in the 14–114 kDa range were distributed in the Mw areas A1 (16.8 ± 0.9%), A2 (79.2 ± 1.3%), and A3 (4.0 ± 2.2%) with predominant peaks at 17.0 and 53.0 kDa. After 14 h of fermentation, in spontaneously fermented SL_CS and in inoculated SL_C 21B, overlapping banding patterns, with respect to t0, were observed: in the former, about 11 peaks/bands in the 14–115 kDa range and a similar peak distribution in Mw areas A1 (20.8 ± 10.9), A2 (76.5 ± 9.5), and A3 (2.8 ± 1.3) were observed, while, in the latter, the proteolysis carried out by the starter strain *Lpb. plantarum* ITM21B resulted, at t14, in the complete degradation of proteins with Mw > 79 kDa, and the banding pattern showed 7 peaks/bands in the 14–60 kDa Mw range distributed in A1 (41.0 ± 14.1%) and A2 (59.0 ± 14.2%). Although, in both SLs, fermentation produced an increase in percentage of low Mw peaks/bands and a concomitant decrease in high Mw peaks/bands; a significant 75% increase in the 16.6 kDa peak/band was observed only in the inoculated SL, while a minor 29% increase was found in SL_CS. Even the decrease in the 52.2 kDa high Mw peak/band was more marked in SL_C 21B (21%) with respect to SL_CS (11%).

**Figure 3:**
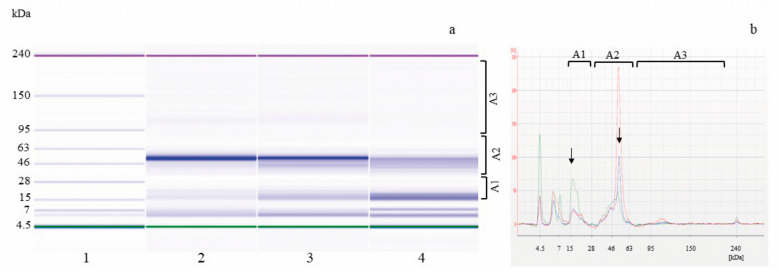
Electrophoretic analysis (LoaC) of total proteins from SL_W inoculated with *W. cibaria* C43-11 (SL_W C43-11) or not (SL_WS), shown as gel-like images (**a**) and overlaid electropherograms (**b**) on Protein230 LabChip. Ladder, weight marker (1), SL_WS t0 (2, red line), SL_WS t14 (3, blue line), and SL_W C43-11 t14 (4, green line). Brackets indicate molecular weight areas: A1 (14–30 kDa), A2 (31–79 kDa), and A3 (80–230 kDa); arrows indicate predominant 21.6 and 50.0 kDa protein peaks/bands.

Figure 3 shows the representative electrophoretic profiles of the total proteins extracted from the SL_W samples containing only hemp flour.

At sampling time t0, protein pattern analysis enabled the observation of about 11–13 peaks/bands with Mw in the 14–108 kDa range distributed in the Mw areas A1 (25.6 ± 8.2%), A2 (70.6 ± 8.6%), and A3 (4.4 ± 1.4%) with predominant peaks at 21.6 and 50.0 kDa. After 14 h of fermentation at 30 °C, in the presence or not of the starter *W. cibaria* C43-11, protein patterns were perfectly superimposable, confirming results of the Bradford quantifications. In spontaneously fermented SL_WS, about 12 bands with Mw from 14 to 100 kDa were observed with a comparable distribution in Mw areas A1 (24.9 ± 3.3%), A2 (71 ± 2.7%), and A3 (4.1 ± 0.6%); in SL_W C43-11, the protein pattern, although presenting an identical profile with about 14 peaks/bands in the 14–102 kDa range matching the predominant peaks/bands, showed a slight increase in peaks/bands in Mw A1 (29.3 ± 7.1%) and a concomitant decrease in higher molecular weight proteins present in A2 (67.4 ± 7.3%) and A3 (3.3 ± 1.2%). Pontonio et al. [57] showed that LAB fermentation at 30 °C for 24 h, using a *Leuconostoc mesenteroides* strain, led to the release of bioactive peptides and an increase in protein digestibility through proteolysis that significantly improved the antioxidant potential of a hemp–water mixture (1:1 ratio). Other researchers [56] found that the highest protease activities in hemp-based fermentation products were found after fermentation with an *Lvb. brevis* strain. Indeed, microbial proteases acting during protein hydrolysis led to the formation of smaller peptides and TFAA and to the formation of intermediates during the synthesis of aromatic products [58]. Dallagnol et al. [59] also reported that after quinoa seed fermentation with LAB, only small proteins (22 kDa) were formed.

As a result of the proteolysis, the TFAA content (Table 5) showed some differences among samples. The sourdoughs produced exclusively with hemp flour and water showed a higher (*p* < 0.05) initial TFAA content in comparison to the formulation containing wheat and hemp flour. While the amount of TFAA did not increase following 14 h of spontaneous fermentation in both formulations, SL_W C43-11_t14 exhibited a TFAA content that was significantly higher with respect to t0, corresponding to an increase of 158% (Table 5). Hemp seed proteins have good amounts of sulfur-containing amino acids, very high levels of arginine and glutamic acid, and consistent digestibility [2]. Other studies [15,57] showed an increase in TFAA content during hemp fermentation by LAB strains. Nionelli et al. [15] observed a relevant concentration of glutamate in uninoculated hemp sourdough before fermentation (235 ± 3 mg/kg), and glutamate (691 ± 6 mg/kg), proline (480 ± 3 mg/kg), alanine (383 ± 5 mg/kg), serine (362 ± 4 mg/kg), and the functional non proteic amino acid GABA (323 ± 3 mg/kg) in hemp sourdough (DY of 160 at 30 °C for 24 h) were found when inoculated with *Lpb. plantarum*/s5, *P. acidilactici*/s5, and *Leuc. meseteroides*/s1 pool strain.

The presence of L-glutamic acid was also monitored since the taste-active features of this compound can be naturally present in cereal matrices, and it is produced by LAB fermentation [19,60]. In fact, the L-glutamic acid content at the beginning of SL fermentation ranged between 0.51 ± 0.09 and 0.79 ± 0.09 mmol/kg. No differences after fermentation were registered.

Other key metabolites deriving from LAB fermentation are exopolysaccharides, including dextran, which are mainly used in the food sector [23,28]. The presence of sucrose in the SL_W formulation favored the production of EPS by the strain *W. cibaria* C43-11 (Table 5), confirming its efficacy as an EPS producer [22]. Meanwhile, the EPS content did not change after spontaneous fermentation, indicating the absence, in the consortia, of microbial strains able to produce EPS starting from sucrose.

An additional parameter monitored after fermentation was the polyphenol content (Table 6). The total polyphenols analysis, as an index of the antioxidant power of the matrix, showed a higher polyphenol content in the SL_W sample than the SL_C sample, because of the higher percentage of hemp flour which contains more polyphenols than wheat flour [61].

The quantification of total polyphenols in sample SL_CS, subjected to the piloted fermentation, highlighted a slight increase in total polyphenols content during fermentation (t0 vs t14); a higher increase (*p* < 0.05) was recorded for the spontaneously fermented SL_C at t14 compared to t0. The total polyphenols content in the sample SL_WS inoculated with the *W. cibaria* C43-11 strain increased 21% after 14 h of fermentation, while for the spontaneously fermented sample, there was no significant total polyphenols increase at t14 compared to t0. Our results demonstrated the influence of the starting cell density on bioactive molecule production during a short fermentation (14 h) since 5 log CFU/g of *Lpb. plantarum* ITM21B did not allow for substantial changes in their content.

Generally, fermentation with LAB strains influences the polyphenols content, leading to the conversion of more complex molecules into simpler ones due to the presence of microbial enzymes such as hydrolases, decarboxylases, and others and an increase in the phenolic stability due to pH lowering [62]. Fermentation activated by Weissella spp. led to increased antioxidant power related to the activity of several enzymes (tannase, decarboxylase, and feruloyl esterase); this results in a release of simpler phenolic compounds with a higher bioavailability [63].

Sourdough production with a shorter time of fermentation (6–24 h) with respect to more traditional (Type I) methods will certainly spread its use at artisanal and industrial levels, given undoubted advantages in terms of sensory, rheology, shelf life, and multiple nutritional attributes. In the current study, the microbial consortia established in wheat–hemp-based sourdough after a short, piloted (*Lpb. plantarum* ITM21B) fermentation enabled the enrichment of the final product, mainly in the content of organic acids and TFAA, as similarly observed in other ITM21B-started sourdough formulations based on different flour types (quinoa, amaranth, and wheat gluten) and can used as taste improvers in salt-reduced breads [18,19] or investigated for EPS production [20]. In the same way, the use of hemp flour in sourdough resulted in a valorization of this matrix by *W. cibaria* C43-11 metabolism.

The ecosystem of a sourdough represents a stressful environment for residing microorganisms, as reported by some authors [53,64,65], and hence, it allows for growth through adaptation of a specific microbiota that express the physiological and stress responses to protect metabolic activities and encourage survival. This could be ascribed to different factors depending on the fermentation conditions and sourdough formulation: (i) the variable carbohydrate and other nutrient concentrations that occur during fermentation time; (ii) the acidic stress (pH of about 4.0) mainly occurring during LAB fermentation; (iii) the variable and limited oxygen concentration caused by the high viscosity relevant to the sourdough dough yield and consistency [10].

### 3.2. Microbial Ecology in SLs

**Figure 4:**
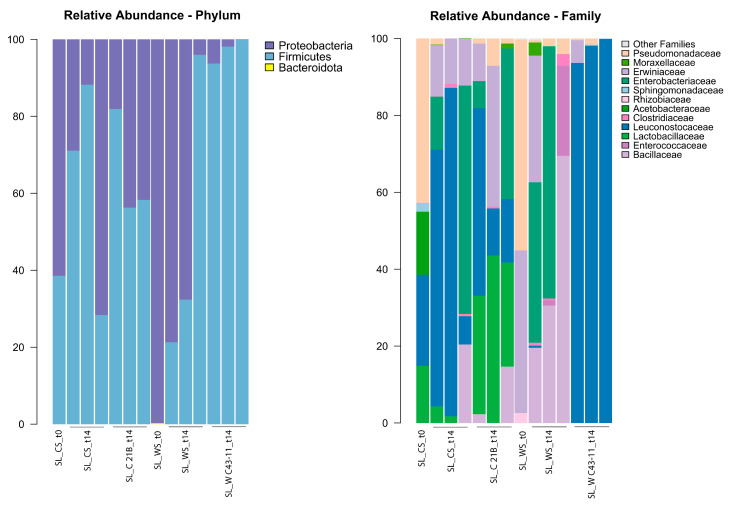
Composition of the microbial communities of the SL formulations and at the various sampling times. Specifically, the relative abundance of all bacterial phyla (on the left) and of the bacterial families with a relative abundance > 0.5% in at least 1 sample (on the right) is represented.

In the current study, it was observed that at the phylum level, the microbial community of SLs was mainly composed of two phyla, *Firmicutes* and *Proteobacteria*. However, *Proteobacteria* represented only one phylum in unfermented sourdough formulations including exclusively hemp seed flour (SL_W t0). The detailed composition of the bacterial community of the SLs at the phylum level is reported in Table 7, and the average percentage of the relative abundance ± SE of the identified phyla and families are shown in Figure 4. Generally, spontaneous flour–water mixture fermentation starts with metabolically active autochthonous, sourdough non-specific bacteria, such as *Proteobacteria*, *Staphylococci*, and diverse LAB species, belonging to the *Enterococci*, *Lactococci*, and *Streptococci* genera, which likely represented the outcome of environmental contamination [10]. This is followed, mainly during the backslopping process, by the growth of autochthonous LAB species, belonging to various species of the *Lactobacillaceae* (encompassing *Lactobacillus* and various genera related to *Lactobacillus*, *Leuconostoc*, *Pediococcus*, and *Weissella*) [10]. All these microorganisms cause a fast acidification of the flour–water mixture inducing the growth (or stabilization) of sourdough-specific LAB species. In particular, microorganisms that occur mainly in spontaneous sourdoughs are *Fructilactobacillus sanfranciscensis* as LAB species and *Kazachstania humilis* as yeast species [64,66,67].

However, the microbial composition expected from the sourdough formulation including solely hemp seed flour or a mixture with wheat flour could be different in spontaneous and in piloted fermentation with a starter LAB strain. The metabarcoding sequencing data provided more details on the populations involved in the fermentation process at the various sampling times, since the microbiome involved in food fermentation, either in the form of starter cultures or as the microbiome of the raw materials in spontaneous fermentations, are spatiotemporally dynamic within the food matrix and vary depending on the flour that composes the sourdoughs. In fact, as reported by Srinivas et al. [68], the complexity of a flour’s mix compared to a basic formulation produces differences in the composition of the microbial communities and consequent complex interactions such as cross-feeding of metabolites produced by one species to another, and/or in competitive or cooperative relationships with one other, that are implicated in patterns and interactions associated with acids and ethanol production, amino acid and sugar metabolism, and lipid and protein lysis.

In the current study, at the level of the bacterial families in the samples, a different composition among each SL formulation was observed. Specifically, for the SL_C formulations, the most abundant families were *Lactobacillaceae* (average relative abundance; SL_CS t0 14.8%; SL_CS t14 2.0%; SL_C 21B t14 33.8%), *Leuconostocaceae* (SL_CS t0 23.7%; SL_CS t14 53.2%; SL_C 21B t14 25.9%), *Acetobacteraceae* (SL_CS t0 16.4%; SL_CS t14 0.05%; SL_C 21B t14 0%), *Enterobacteriaceae* (SL_CS t0 0%; SL_CS t14 24.4%; SL_C 21B t14 15.4%), *Erwiniaceae* (SL_CS t0 0%; SL_CS t14 12.4%; SL_C 21B t14 15.5%) and *Pseudomonadaceae* (SL_CS t0 42.7%; SL_CS t14 0.5%; SL_C 21B t14 3.2%). Among the SL_W formulations, a very different bacterial community was observed. In detail, the SL_WS t0 sample had *Erwiniaceae* (42.3%) and *Pseudomonadaceae* (54.82%) as the most abundant families; for SL_WS t14, the most abundant bacterial families were *Bacillaceae* (39.9%) and *Enterobacteriaceae* (35.8%); and finally, SL_W C43-11 at t14 was made up, as expected, almost exclusively of the *Leuconostocacae* bacterial family (97.2%), since it was inoculated using a strain (*W. cibaria* C43-11) belonging to the *Leuconostocaceae* family.

The bacterial sequences from the total DNA assigned to bacterial phyla and their relative abundances (%) varied depending on the flour composing the sourdoughs. It is interesting to highlight that the unfermented flour–water mixture (t0 samples) contained two main bacterial families with a relative abundance > 40%, namely the *Pseudomonaceae* and *Erwinaceae* families, with the latter present only in the sourdoughs based exclusively on hemp flour. Our results showed that spontaneous fermentation of the SL_WS formulation resulted in the growth (and stabilization) of Bacillaceae (Firmicutes) and *Enterobacteriaceae* (*Proteobacteria*). Also, Ercolini et al. [69] found, when studying the bacterial ecology during rye and wheat sourdough preparation, that by backslopping for 5 to 7 days, Firmicutes became dominant after 1 day of propagation and Weissella spp. were dominant in rye flour and sourdoughs.

Indeed, culture-independent approaches, such as metabarcoding, represent a powerful tool to study the microbiota dynamics along the fermentation process of hemp sourdough, especially in spontaneous fermentation. With respect to culture-dependent methods, the metabarcoding sequencing data provide more details on the populations involved in the fermentation process along the sampling times, since microbiota involved in food fermentation, either in the form of starter cultures or as the microbial community of the raw materials in spontaneous fermentations, are spatiotemporally dynamic within the food matrix and vary depending on the flour that composes the sourdoughs. An accurate identification of the core microorganisms involved in the fermentation, their metabolic capabilities, and the potential synergistic cooperations represent the main aspects to be investigated within the perspective of reconstituting a possible synthetic community that closely resemble spontaneous fermentation, tailored to the needs of different consumers [13,70].

Culture-dependent approaches showed that in *T. durum* grains, LAB were naturally present at low levels (ca. 2.0 log CFU g^−1^), and their population mainly consisted of *Enterococcus, Lactobacillus*, *Lactococcus*, *Weissella*, and *Pediococcus* genera [71]; Cannabis sativa seed flour constituted a population mainly of *Pediococcus*, *Leuconostoc* and *Enterococcus* genera [14]. Hence, the *Lpb. plantarum* ITM21B strain used to pilot the fermentation resulted in different population distributions versus the spontaneous fermentation, mainly represented by *Firmicutes* (97.2%, Table 7), especially the families *Lactobacillaceae* and *Leuconostocaceae*, due to their ability to growth and survive in acidic conditions. A formulation of SL_C was used in this study since a previous investigation [19] demonstrated that the liquid sourdough obtained using a 1:1 w/w ratio of wheat and wheat gluten flour with *Lpb. plantarum* ITM21B as a starter positively affected the sensorial and textural properties of the resulting salt-reduced bread. Moreover, the in silico simulations of growth and organic acid kinetics of *Lpb. plantarum* ITM21B reported by Di Biase et al. [21] suggested that the fermentation process of sourdough based on wheat and wheat gluten flours, using a low inoculum load (4 or 5 log CFU/g), a temperature of 37 °C, and an overnight fermentation time (about 14–17 h), could be sustainably used to enrich the sourdough in organic acids. Our sourdough formulation SL_C ITM21B (fermented for 14 h at 37 °C using a cell density of 5 log CFU/g) positively affected the content of bioactive molecules (polyphenols, organic acids, and amino acids).

On the other hand, the *W. cibaria* C43-11 strain used to pilot the fermentation, starting with an initial cell density of 8.00 ± 0.0 CFU/g of SL, became dominant after the short fermentation, while other populations, including *Enterobacteriaceae*, were almost completely inhibited. Indeed, this strain is known to have remarkable ecological advantages in carbohydrate-rich environments due to its rapid sugar fermentation and production of antimicrobial compounds such as bacteriocins [71]. Furthermore, the lactic acid production in SL_W C43-11 causes a reduction in the environmental pH and the consequent inhibition of competing microbial species, while its resistance to acid and osmotic stress enhances its survival in competitive niches, such as fermented food environments [71]. Also, in a previous study, the impact of the microbial starter *Leuconostoc citreum* C2.27 on the composition of the dough microbiota was investigated by both a culture-dependent approach and a metagenomic analyses [54], revealing how selected starters could reshape the dough microbiota. Knowledge of the composition of microbial communities is important for understanding and eventually steering their functionality [72].

**Figure 5:**
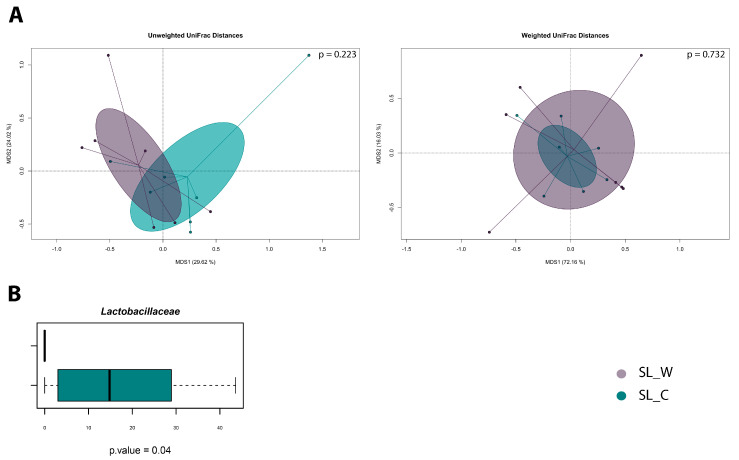
(**A**) PCoA plots based on unweighted and weighted UniFrac distances. (**B**) Boxplots representing the variation in relative abundance between the SL_C and SL_W formulations. Kruskal–Wallis test results with a *p*-value lower than 0.05 were highlighted under the boxplot. The color legend is represented in the bottom-right corner of the figure.

Also, focusing attention on the general microbial layout of the two studied formulations, we observed no significant differences between the two microbial communities, as observed by the two PCoA plots in Figure 5A (Test adonis with pseudo-F ratio, *p* > 0.05). However, in relation to the point variation at the family level, the SL_C samples showed a significant enrichment of the *Lactobacillaceae* family compared to the SL_W samples (Kruskal–Wallis test, *p* = 0.04; Figure 5B).

## 4. Conclusions

The data obtained in the current study suggest that the dynamic of microbial populations during the fermentation process were mainly determined by microbiota native to the raw material. Thereafter, the composition and bioavailability of the nutrients characteristic of the flours used in this study and their modification occurring after acidification promote the production of various compounds in the sourdough fermentation process. As reported in the Result Section, the hemp, either in the hemp-based sourdough or in the wheat–hemp mixture sourdough, enabled the obtainment of a microbial composition that favored the production of organic acids, total polyphenols, and total free amino acids (TFAAs). Moreover, the ability of the *W. cibaria* C43-11 strain to produce dextran was observed in sourdough formulated exclusively with the water and hemp flour mixture in the presence of sucrose as the additional carbon source. However, these preliminary results pointed out the need for more in-depth investigations in order to isolate and better characterize the key players of the fermentation steps mainly responsible for these metabolic activities. In this respect, studying the competitive or cooperative relationships among microbial populations and the effects on nutritional features characterizing hemp-based liquid sourdough will contribute to the selection of the most effective and the best performing consortia from a complex microbiota composition providing new insights into the creation of reconstituted synthetic communities. Accordingly, the microbial ecology dominating the sourdough formulations started by *W. cibaria* C43-11 and *Lpb. plantarum* ITM21B represents two examples of consortia with possible future applications in manufacturing fermented food ingredients to be used as fat or salt substitutes in baked goods.

## Figures and Tables

**Table 1:** Formulation of SLs and starting fermentation conditions.

	SL_C		SL_W	
SL composition *	SL_C 21B	SL_CS not inoculated	SL_W C43-11	SL_WS not inoculated
Wheat flour type 0	50 g		-	
Hemp flour	50 g		100 g	
Sucrose	-		15 g (15% w/fw)	
Tap water	150 mL		150 mL	
Dough yield (DY)	250		250	
	Starting conditions			
Temperature (°C)	T: 37 °C	T: 37 °C	T: 30 °C	T: 30 °C
Starter	*Lpb. plantarum* ITM21B	-	*W. cibaria* C43-11	-
(N_0_) log CFU/g	5 log CFU/g	-	8 log CFU/g	-
pH	6 ± 0.5	6 ± 0.5	6 ± 0.5	6 ± 0.5
Sampling times (h)	t0; t14	t0; t14	t0; t14	t0; t14

Formulations and fermentation conditions were established in previous [19,20,21,22] and ongoing studies. * SL_C formulation was prepared with wheat–hemp flour mixture (1:1) and analyzed before (t0) and after (t14) spontaneous (SL_CS) or piloted (SL_C 21B) fermentation; SL_W formulation was prepared with hemp flour (100%) and analyzed before (t0) and after (t14) spontaneous (SL_WS) or piloted (SL_W C43-11) fermentation.

**Table 2:** Values of pH, TTA, and LAB count in liquid sourdoughs.

	**SL_CS_t0**	**SL_CS_t14**	**SL_C 21B_t14**
pH	6.98 ± 0.01 a	6.12 ± 0.65 b	5.73 ± 0.03 b
TTA (mL)	0.97 ± 0.06 a	3.80 ± 1.99 b	5.83 ± 1.16 c
LAB (log CFU/g)	2.54 ± 0.01 a	7.27 ± 2.10 b	7.55 ± 0.63 b
	**SL_WS_t0**	**SL_WS_t14**	**SL_W C43-11_t14**
pH	7.18 ± 0.08 a	6.76 ± 0.01 b	5.31 ± 0.20 c
TTA (mL)	1.07 ± 0.06 a	2.00 ± 0.00 b	11.13 ± 0.55 c
LAB (log CFU/g)	2.54 ± 0.01 a	6.35 ± 0.39 b	8.27 ± 0.11 b

S_t0 samples were unfermented; S_t14 samples were spontaneously fermented; Strain_t14 samples were fermented with starter strain addition. Values (mean ± standard deviation) with different lower-case letters in the same row are significantly different (*p* < 0.05).

**Table 3:** Organic acids (lactic, acetic, and propionic) quantified in the liquid sourdoughs.

		Organic Acids Content (mmol/kg ± SD)		
		Lactic	Acetic	Propionic
SL_C	SL_CS t0	<DLa	0.22 ± 0.34 a A	0.43 ± 0.13 a A
	SL_CS t14	1.99 ± 1.73 b	0.70 ± 0.27 ab	0.39 ± 0.0 a
	SL_C 21B t14	9.12 ± 1.22 c	2.30 ± 1.64 b	<DLb
SL_W	SL_WS t0	<DLa	2.51 ± 0.57 a B	1.23 ± 0.20 a B
	SL_WS t14	<DLa	2.15 ± 0.97 a	1.07 ± 0.37 ab
	SL_W C43-11 t14	7.47 ± 0.75 b	2.26 ± 0.27 a	0.75 ± 0.14 b

S_t0 samples were unfermented; S_t14 samples were spontaneously fermented; Strain_t14 samples were fermented with starter strain addition. Values (mean ± standard deviation of the mean) with different lower-case letters in the same column for each acid are significantly different among the same formulation (*p* < 0.05) for SL_C or SL_W. Values with different capital letter in the column are significantly different (*p* < 0.05).

**Table 4:** Total protein content in liquid sourdoughs formulations.

SL Formulation	Total Protein Content (g/500 g of SL ± SD)		
SL_C	**SL_CS_t0**	**SL_CS_t14**	**SL_C 21B_t14**
	17.26 ± 0.01 a	14.78 ± 0.77 b	14.97 ± 0.56 ab
SL_W	**SL_WS_t0**	**SL_WS_t14**	**SL_W C43-11_t14**
	11.06 ± 0.02 a	12.62 ± 0.26 a	11.56 ± 0.20 a

S_t0 samples were unfermented; S_t14 samples were spontaneously fermented; Strain_t14 samples were fermented with starter strain addition. Values (mean ± standard deviation) with different lower-case letters in the same row are significantly different (*p* < 0.05).

**Table 5:** TFAA and EPS content in unfermented (t0) and fermented SL samples.

	TFAA mg/kg ± SD	EPS g/kg ± SD
SL_CS_t0	285.3 ± 28.7 a A	
SL_CS_t14	283.3 ± 57.0 a	
SL_C 21B_t14	388.8 ± 92.4 a	
SL_WS_t0	417.2 ± 97.6 a B	0.34 ± 0.09 a
SL_WS_t14	482.3 ± 10.1 a	0.90 ± 0.71 a
SL_W C43-11_t14	1075.9 ± 399.5 b	15.29 ± 1.53 b

S_t0 samples were unfermented; S_t14 samples were spontaneously fermented; Strain_t14 samples were fermented with a starter strain addition. Values (mean ± standard deviation) with a different lower-case letter in each column are significantly different among the same formulation (*p* < 0.05) SL_C or SL_W. Values with a different capital letter in the column are significantly different (*p* < 0.05).

**Table 6:** Total polyphenol concentrations (mg GAE/100 g of SL) in methanol extract of liquid sourdoughs.

Total Polyphenols (mg GAE/100 g of SL)		
**SL_CS_t0**	**SL_CS_t14**	**SL_C 21B_t14**
59.41 ± 1.08 a A	63.87 ± 0.72 b	62.42 ± 2.24 ab
**SL_WS_t0**	**SL_WS_t14**	**SL_W C43-11_t14**
96.61 ± 2.40 a B	92.25 ± 4.69 a	117.24 ± 3.02 b

GAE: Gallic Acid Equivalent. S_t0 samples were unfermented; S_t14 samples were spontaneously fermented; Strain_t14 samples were fermented with a starter strain addition. Values (mean ± standard deviation) with different lower-case letters in a row are statistically different among the same formulation (*p* < 0.05), n = 3. Values with a different capital letter in the column are significantly different (*p* < 0.05), n = 3.

**Table 7:** Relative abundance values of the phyla present in the microbial community of the SL samples.

	**SL_CS t0**	**SL_CS t14**	**SL_C 21B t14**
*Bacteroidota*	0.0	0.0	0.0
*Firmicutes*	38.6%	62.5% ± 17.8%	65.5% ± 8.2%
*Proteobacteria*	61.4%	37.5% ± 17.8%	34.5% ± 8.2%
	**SL_WS t0**	**SL_WS t14**	**SL_W C43-11 t14**
*Bacteroidota*	0.3%	0.0	0.0
*Firmicutes*	0.0	49.9% ± 23.3%	97.2% ± 1.8%
*Proteobacteria*	99.7%	50.1% ± 23.3%	2.8% ± 1.8%

S_t0 samples were unfermented; S_t14 samples were spontaneously fermented; Strain_t14 samples were fermented with starter strain addition.

## Data Availability

The original contributions presented in this study are included in the article. Further inquiries can be directed to the corresponding author. On the submission system.
